# Does the use of National Early Warning Scores (NEWS or NEWS2) in healthcare settings improve patient outcomes: a systematic review

**DOI:** 10.1186/s13643-026-03088-y

**Published:** 2026-01-31

**Authors:** Lauren J. Scott, Joni Jackson, Sarah Dawson, Thomas Knight, Jelena Savović

**Affiliations:** 1 National Institute for Health and Care Research Applied Research Collaboration West (NIHR ARC West), University Hospitals Bristol and Weston NHS Foundation Trust, Bristol, UK; 2 Population Health Sciences, Bristol Medical School, University of Bristol, Bristol, UK; 3 Division of Immunology, Immunity to Infection and Respiratory Medicine, University of Manchester, Manchester, UK

**Keywords:** National Early Warning Score, Physiology, Patients, Clinical outcomes, Systematic review

## Abstract

**Background:**

The National Early Warning Score (NEWS), and updated NEWS2, is used to detect all-cause deterioration, based on clinical observations. Many studies have validated the prognostic accuracy of NEWS/NEWS2, but few have investigated whether its use improves patient outcomes. This systematic review aims to bring together all evidence evaluating the effectiveness of using NEWS/NEWS2, compared to not using NEWS/NEWS2, on patient outcomes.

**Methods:**

We searched Embase, MEDLINE, CINAHL, The Cochrane Library, and international trial registries from January 2012 (when NEWS was developed) to October 2024, for all records which mentioned NEWS/NEWS2 in their title or abstract. All comparative studies with interventions which included NEWS/NEWS2, and comparator areas, regions, organisations, time points, or settings which did not, were included. The target population was all non-maternity patients aged 16+ years treated in healthcare settings. Where possible, studies were synthesised by outcome using random-effects meta-analyses. Risk of bias was assessed using ROB2 and ROBINS-I v2; certainty of evidence was assessed using GRADE. PROSPERO registration CRD42023442061.

**Results:**

We screened 2814 records and included 20 studies (in 32 records), with 18 presenting data for our outcomes. Sample sizes ranged from 66 to 13,059,865 participants. There was low certainty evidence that using NEWS/NEWS2 reduced in-hospital mortality (summary odds ratio [OR] from eleven studies 0.79, 95% confidence interval [CI] 0.66 to 0.94). There was also a little low certainty evidence of a reduction in hospital length of stay (two of four studies) and cardiac arrests (three of seven studies), and a potential reduction in intensive care admissions (summary OR from seven studies 0.63, 95% CI 0.36 to 1.09). Most included studies had a serious/high (12/18; 67%) or moderate (5/18; 28%) risk of bias.

**Conclusions:**

This review highlights a lack of large scale, high-quality studies exploring the effectiveness of NEWS/NEWS2. No included studies reported negative clinical impacts of using NEWS/NEWS2 on any outcomes presented in this review. However, there is still further work required to ascertain whether the use of NEWS/NEWS2 improves patient outcomes.

**Systematic review registration:**

PROSPERO CRD42023442061

**Supplementary Information:**

The online version contains supplementary material available at 10.1186/s13643-026-03088-y.

## Background

Early warning scores (EWS) are track-and-trigger systems which have been used in hospitals since the 1990’s to monitor patients, recognise patient deterioration, and trigger an appropriate clinical response [1]. EWS are calculated based on physiological observations, and different hospitals have historically used a number of slightly different scoring systems.

To align scoring across the country, in 2012, the Royal College of Physicians (RCP) developed the National Early Warning Score (NEWS) as a standarised tool to detect all cause deteroration in hospital patients [2]. It is a clinical scoring system which combines respiratory rate, oxygen saturation, systolic blood pressure, heart rate, temperature, and level of consciousness (using the AVPU scale) to calculate an overall clinical acuity score. The updated NEWS2 was introduced in 2017, adding a new oxygen saturation scale for patients with hypercapnic respiratory failure and amending the level of consciousness scoring to include points for new onset confusion alongside voice, pain, and unresponsiveness [3]. NEWS and NEWS2 values lie between 0 and 20, with higher scores suggesting higher clinical acuity and poorer outcomes [3]. NEWS2 is now mandated by NHS England for use in the ambulance service and acute hospitals in England and is also recommended for use across the wider NHS (for example in primary care) as a common language [4, 5].

Since its inception in 2012, there have been a huge number of studies which have validated the prognostic accuracy of NEWS and subsequently NEWS2 (referred to collectively throughout this paper as NEWS/NEWS2) in different healthcare settings [6–13], and most of these agree that it is a useful tool for the detection of patient deterioration, at least in the short term. However, to date, there have only been a small number of studies which have investigated whether the use of these scores improves clinical outcomes such as mortality and ICU admissions, and most of these are uncontrolled before-after studies.

This systematic review aims to bring together all the evidence to date evaluating the effectiveness of using NEWS/NEWS2, compared to not using NEWS/NEWS2, on patient outcomes including mortality, hospital admissions, ICU admissions, hospital length of stay, sepsis, suspicion of sepsis, and cardiac arrests.

## Methods

The study protocol was registered on PROSPERO (CRD42023442061) [14]. Results are reported according to PRISMA guidelines [15].

### Eligibility criteria

*Study design*: We included all randomised trials and non-randomised intervention studies such as cohort studies, controlled or uncontrolled before-after studies (i.e. studies which use historical controls), and interrupted time series studies. Both cluster and individually allocated studies were eligible.

*Participants*: The target population included all non-maternity patients aged 16+ years treated in healthcare settings such as hospitals or primary care.

*Interventions and comparators*: We included all studies where the intervention included the use of NEWS or NEWS2 and compared this to areas, regions, organisations, time points, or settings where NEWS/NEWS2 was not in use. Studies which investigated NEWS/NEWS2 for which the included parameters were modified in any way (e.g. NEWS + lactate) were excluded.

*Outcomes*: The primary outcome for this study was mortality; we did not pre-specify a particular time frame for this, and as such include mortality across any time period. Pre-defined secondary outcomes were sepsis, suspicion of sepsis, mortality due to sepsis, mortality due to suspicion of sepsis, hospital admissions, and length of hospital stay. We also report cardiac arrests and intensive care unit (ICU) admissions as post-hoc secondary outcomes as these were outcomes in several of our included studies. Studies which met all other inclusion criteria but did not present any of our outcomes of interest were included in the review but not included in quantitative synthesis.

*Publication type*: All publication types including peer-reviewed papers and conference abstracts were eligible.

### Literature search

We searched Embase, MEDLINE, CINAHL, The Cochrane Library, and international trial registries (ClinicalTrials.gov and the WHO International Clinical Trials Registry Platform) from January 2012 (when NEWS was developed by the RCP) to October 2024, for all records which mentioned NEWS/NEWS2 in their title, abstract, author assigned key words, or database subject headings. We also searched for existing systematic reviews with the broader concept of early warning scores/systems and/or track and trigger systems. The search terms were developed by an information specialist (SD) in liaison with the rest of the team and were adapted for each database (Supplemental material). We supplemented these searches by examining the reference lists of our included studies, and by searching for papers which have referenced our included studies. There were no language restrictions; however, as NEWS/NEWS2 was developed in the UK, included studies from outside the UK were carefully checked to make sure they were referring to the NEWS/NEWS2 scoring system as developed by the RCP.

### Study selection

Identified titles and abstracts were independently screened by two reviewers (LS and JJ). Full text papers were obtained for all abstracts that were included by at least one reviewer. Full texts were independently screened against the inclusion criteria by the two reviewers, with any discrepancies resolved via consensus. Percentage agreement for full text screening is reported along with a Cohen’s Kappa statistic.

### Data extraction

Titles and abstracts were screened using the Rayyan web platform [16]. Full texts were screened using Endnote bibliographic software [17]. A data extraction form was developed in Microsoft Excel. Data were extracted on study design and location, population characteristics, interventions and outcomes studied, and study results. Data extraction was carried out by one reviewer and checked by another. When necessary, authors were contacted for further information or clarification.

### Risk of bias assessments

Risk of bias for each included outcome was assessed using the Cochrane risk of bias tool RoB2 [18] for (cluster) randomised controlled trials (RCTs) and ROBINS-I [19] v2 for non-randomised studies. The comparison of interest was assignment to the intervention (i.e. intention to treat) for all risk of bias assessments. Assessments were carried out by two reviewers, and discrepancies were resolved through discussion.

### Data synthesis

We narratively summarised the data from all included studies, grouped by outcome. Where appropriate and possible, meta-analyses were carried out to combine results. Primary analyses used random-effects meta-analyses, due to anticipated heterogeneity between studies (e.g. setting and population), with fixed-effects models provided as sensitivity analyses (with both estimates shown on the same forest plot). The *meta summarise* and *meta forestplot* commands in Stata version 18.0 were used to calculate and present effect estimates, 95% confidence intervals (CIs), *p*-values, and heterogeneity (*I*^2^). Odds ratios (ORs), incidence rate ratios (IRRs), and hazard ratios (HRs) are presented for dichotomous outcomes, and mean differences (MDs) are presented for continuous outcomes. Where possible, adjusted estimates are presented; however, for studies where only raw data or unadjusted estimates have been reported, we present unadjusted estimates. For outcomes with zero events in one cell, 0.5 was added to all four cells in order to calculate an unadjusted odds ratio.

### Subgroup analyses

As proposed in the protocol, we present subgroup analyses by study design (cluster-RCT vs. non-randomised comparative study vs. uncontrol before-after) for all meta-analysed outcomes. We also present post hoc subgroup analyses by intervention type (NEWS alone vs. NEWS as part of a package vs. NEW2 alone). We stated in the protocol we might investigate subgroups by setting or type of patient if appropriate. However, given that all studies were in hospital settings, and there were no patient groups of obvious interest, we did not pursue these subgroup analyses.

### Sensitivity analyses

As conference abstracts are often limited in the data they provide and have a higher risk of bias than peer-reviewed publications, we performed sensitivity analyses excluding studies which presented data in conference abstracts only.

### Certainty of evidence

We rated the certainty in the overall body of evidence by applying the Grading of Recommendations Assessment, Development and Evaluation (GRADE) framework for our synthesised outcomes [20]. We followed the revised GRADE guidelines which propose that when using ROBINS-I tool to assess risk of bias, non-randomised studies can start at ‘high certainty’ (same as RCTs) and be downgraded for identified limitations [21]. We used the GRADE-Pro web application to construct our summary of findings table [22].

## Results

**Fig. 1:**
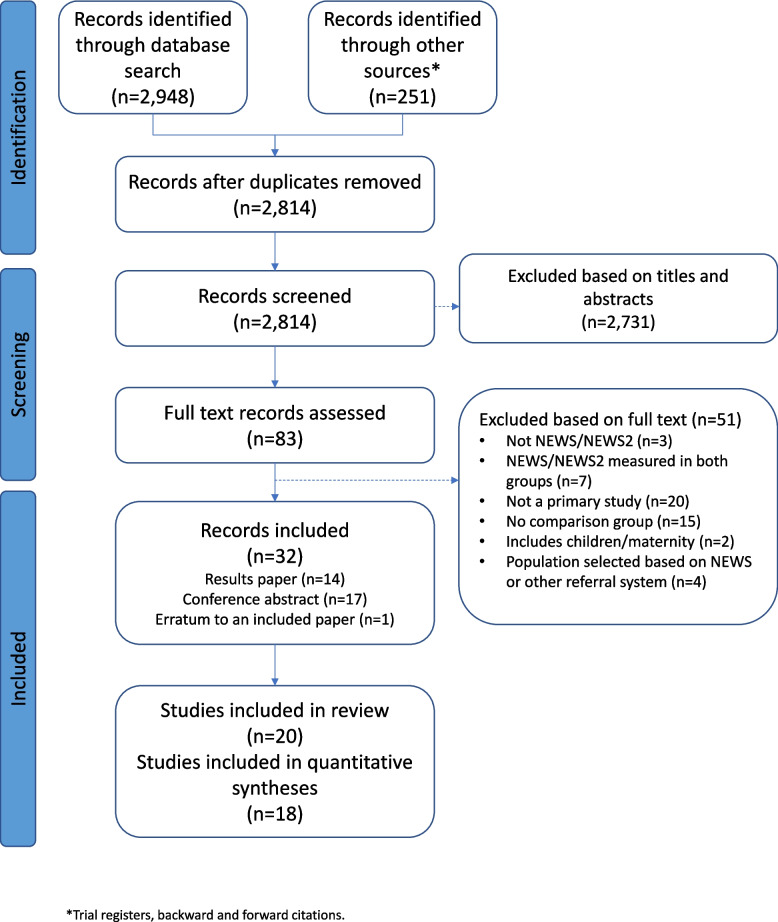
Study flowchart

After removing duplicates, our searches identified 2814 studies. During title and abstract screening, 2731 were excluded leaving 83 for full text screening. Of these, 51 were excluded after full text screening (see Fig. 1 for details). Initial reviewer agreement for full text screening was 92% (Cohen’s Kappa=0.831). We included 20 studies, reported in 32 records [23–54]. Of the 20 primary study reports (i.e. the main study publication or the report with the most relevant data), 12 were research publications and 8 were conference abstracts. There were 17 uncontrolled before-after studies [23, 25, 27–30, 36, 39–41, 43–46, 48, 51, 52], 1 cluster-RCT [53], 2 non-randomised comparative studies [31, 34]. 11 studies investigated NEWS on its own, 7 investigated NEWS as part of an intervention package, 2 investigated NEWS2 on its own, and 0 investigated NEWS2 as part of an intervention package. For all studies, the comparison group was some form of standard care without the use of NEWS/NEWS2.

### Summary of included studies

Characteristics of all 20 included studies are presented in Table 1. Five studies were conducted in the USA, two in the UK, two in Brazil, two in Korea, and one in Bangladesh, Belgium, People’s Republic of China (China), Denmark, Egypt, Italy, Japan, Republic of China (Taiwan), and Thailand. All twenty studies were in hospital settings including: ten were inpatient wards, two emergency departments (ED), one high dependence unit, one all hospital wards, one inpatients on a cardiology ward, one inpatients following cardiac arrest, one patients with suspicion of sepsis, one ED patients with a sepsis diagnosis, one post-haematopoietic stem cell transplantation (HSCT) patients in sterile room unit, and one patients receiving allogeneic and autologous transplantations for hematologic malignancies and cellular therapeutics. Sample sizes ranged from 66 to 13,059,865 participants.

**Table 1:** Study characteristics

First author/PI (year)	Country	Sample size	Setting	Intervention	Intervention details	Control details	Inclusion	Exclusion	Outcomes included in review
**Cluster RCTs:**									
Haegdorens (2018) [53]	Belgium	69,656	Hospital (inpatients)	NEWS as part of package	A standardised observation and communication protocol using the NEWS and SBAR communication method. Hospitals integrated the NEWS in their own paper based or electronic patient record. One week before the start of the intervention, the ward nurses received an interactive 4-h training concerning the measurement and interpretation of vital signs, clinical observation, communication skills, and practical tips and tricks in handling NEWS and SBAR.	Standard care	Patients admitted to the participating wards within the study period were included.	Patients were excluded if they were pregnant or below 17 years of age.	Unexpected in-hospital mortality Unplanned ICU In-hospital cardiac arrests
**Non-randomised comparative studies:**									
Fu (2017) [31]	China	66	Hospital (post-haematopoietic stem cell transplantation (HSCT) patients in sterile room unit)	NEWS as part of package	Patients in the intervention group had a local routine protocol treatment and were further evaluated with NEWS and RRT was activated simultaneously. RRT was activated when total NEWS score exceeded 7 or individual item score exceeded 3.	Standard care	All post HSCT adult patients in sterile room unit who developed persistent fever for 3 days	Not stated	Mortality (90 day)
Hogan (2020) [34]	UK	13,059,865	Hospital (inpatients)	NEWS	Hospitals were categorised as either using NEWS (which included both original NEWS and NEWS to which a limited number of extra items (most commonly urine output) had been added locally or non-NEWS track and trigger system (TTS)	Standard care	All HES patients	Not stated	In-hospital mortality In-hospital cardiac arrests
**Uncontrolled before-after studies:**									
Anam (2023) [23]	Bangladesh	11,008	Hospital (high dependency unit)	NEWS2	Before commencing the Quality Improvement Project (QIP), all involved were taught about the benefits and utility of NEWS2 and trained on its effective application through informal lecture presentations and bedside hands-on training sessions, planned and conducted by the project leader (PL).	Standard care	All adult (age ≥18 years) patients of both genders admitted at the HDU	Those declared as ‘Do Not Intubate/Do Not Resuscitate (DNI/DNR)’, deemed equivalent to ‘Do Not Attempt Cardiopulmonary Resuscitation’ (DNACPR)	In-hospital mortality due to sudden cardiac arrest ICU admission
Badr (2021) [25]	Egypt	364	Hospital (inpatients)	NEWS	The researcher trained the emergency nurses on using NEWS for two months utilising lecture, group discussion, and clinical scenarios. The program was designed to help understand vital signs’ physiological parameters, reasons for measurement and abnormalities, and establish a communication framework between the health care members. It covered the following learning topics: benefits of NEWS, six Physiological Parameters included, outline how NEWS works, threshold and triggers, and demonstrating correct use of NEWS and its clinical response.	Standard care	All adult patients admitted to the studied unit.	Under 18 years old, pregnant women, and patients readmitted to the unit during the study.	Unexpected in-hospital mortality Unplanned ICU
Bedoya (2019) [27]	USA	85,322	Hospital (inpatients)	NEWS	For patients hospitalised on intermediate/non-ICU wards, a best practice advisory (BPA) was triggered to a patient’s care nurse if the NEWS reached a threshold of 7 or higher. Care nurses and nursing leadership were trained on the model parameters and BPA-triggered workflows during educational conferences, online learning modules and live demonstrations. After roll-out of the BPA, regular meetings were held with nursing staff to reinforce appropriate BPA response.	Standard care	All adult (aged ≥18 years) inpatient admissions during the study period	None.	In-hospital mortality ICU admission
Berger (2018) [28]	USA	12,018	Hospital (all hospital patients)	NEWS as part of package	The National Early Warning Score (NEWS) was used to identify patients at high risk (≥7) of adverse outcomes. The automated system continuously displayed updated vital signs, laboratory data, bundles of care and NEWS to all members of the care team.	Standard care	Not stated	Not stated	In-hospital mortality ICU admission Hospital length of stay
Creutzburg (2021) [29]	Denmark	938	Hospital (inpatients who suffered an in hospital cardiac arrest)	NEWS	The NEWS system has an algorithm for the clinical response by the ward staff and when to active the trigger part of the system according to the measured NEWS.	Standard care	All cardiac arrest team activations in the study period, in patients aged ≥18 years.	Patients admitted to wards not using the NEWS system such as intensive care units, operating theatres, cardiac catherization labs, etc.	In-hospital cardiac arrests. 30-day mortality following cardiac arrest
Flato (2021) [30]	Brazil		Hospital (inpatients)	NEWS as part of package	A rapid response team (RRT) assistance protocol was used; that is, a team of intensivists was activated according to the yellow and blue code using the SBAR communication tool and NEWS tool for the early recognition of the patient’s deterioration.	Standard care	Not stated	Not stated	In-hospital mortality In-hospital cardiac arrests
Howard (2022) [36]	USA	19,513	Hospital (emergency department patients)	NEWS as part of package	If and when a vital sign(s) deviation occurred to the point that an overall NEWS score of ‘5’ was reached, an electronic text alert was sent the pagers of both the charge nurse and ED physician, prompting the performance of a rapid clinical assessment. The text of the alert was simply ‘NEWS Alert Score of X, Bed XX.’ Prior to initiation of this system, both nursing and physician staff were trained on the notification process, the clinical significance of the NEWS scores, and expected action after alerts.	Standard care	Not stated	Trauma patients, children and pregnant women	In-hospital mortality Hospital length of stay
Hwang (2022) [39]	Korea	388	Hospital (inpatients)	NEWS2	First, head nurses in the study wards were trained during an hour-long session on NEWS2, including NEWS2 calculation, score-dependent responses, and a checklist to confirm clinical concerns and calculate NEWS2. Next, the trained head nurses educated nurses in small groups in their wards, including the use of shift handover time. For consistent nurse education, we explained the study protocol to head nurses and provided standardised educational material to ensure accurate delivery of the content.	Standard care	Patients (19+ years and hospitalisation length of 2+ days) discharged from the wards during the three months before and after NEWS2 use.	Not stated	Unexpected in-hospital mortality Unplanned ICU In-hospital cardiac arrests
Kim (2019) [40]	Korea	73,927	Hospital (inpatients at a tertiary hospital)	NEWS	NEWS calculated automatically by electronical medical records	Standard care	Age over 18	Intensive care unit and emergency room were excluded	In-hospital cardiac arrests requiring CPR
Kuo (2017) [41]	Taiwan	181,169	Hospital (inpatients at a tertiary hospital)	NEWS as part of package	The key interventions included electronic NEWS, nurses and physicians computer-based reminding alarm if NEWS≥ 7 or more than highest scores among previous 3 measurements, real time early warning screen saver and electric board, in service education and early warning monitor team.	Standard care	Not stated	Not stated	In-hospital cardiac arrests
McCoy (2023) [43]	USA	703	Hospital (emergency department patients with a sepsis diagnosis)	NEWS	The protocol used triage NEWS≥7 to trigger a code sepsis like trauma and 5–6 to alert providers verbally and via a chart sticker to potential sepsis.	Standard care	Adults >21 years with a final ED diagnosis of sepsis and all variables available.	Not stated	In-hospital mortality Hospital length of stay
Novelli Oliveira (2022) [44]	Brazil	280	Hospital (emergency department patients)	NEWS	The use of NEWS including the recommendation of the time interval for the subsequent evaluation: category zero—every 12 h; category one to four—between 4 and 6 h; category five or more, or score three on one of the parameters—every hour; and category seven or more—continuous monitoring of vital signs. Nursing teams were trained according to the guidelines of the RCP manual updated in 2017. During the meetings, the following aspects of the NEWS preparation program were addressed: the importance of vital signs for the identification of early clinical deterioration in the emergency room; the characteristics of NEWS and their relationship with the monitorization of vital signs; and the presentation of the use of NEWS, in addition to the exercise of applying the NEWS in a hypothetical case typical of the emergency room, including its documentation.	Standard care	Patients aged ≥18 years, admitted to one of the ten beds with multiparametric monitorization in the emergency room participated in the study.	Patients whose medical records were illegible or erased and patients who evaded or were in palliative care were excluded. Patients admitted more than once during the study period were included only on their first admission to the unit.	None
Pullyblank (2020) [45]	UK	Not stated	Hospital (patients with suspicion of sepsis codes)	NEWS	Many interventions were tested and then implemented by different organisations to aid adoption of NEWS. These included acute trusts, community, digital enablers, education and training, and patient and public involvement.	Standard care	Patients with suspicion of sepsis diagnosis codes	Not stated	Mortality
Spagnolli (2015) [46]	Italy	2,100	Hospital (inpatients)	NEWS as part of package	An organisational model differentiated by intensity of care (IC), where patient-bed assignment to areas of the ward, was based on clinical assessment and National Early Warning Score (NEWS)	Standard care	Not stated	Not stated	Mortality (72 h) Unplanned ICU
Suhr (2020) [48]	USA	410	Hospital (patients receiving allogeneic and autologous transplantations for hematologic malignancies and cellular therapeutics)	NEWS	The bedside nurses were responsible for manually calculating the NEWS score and implementing the correct patient care response, such as activating a rapid response team (RRT). A score of ≤4 requires no intervention. A score of 5–6, or a single parameter score of 3, requires communication with the physician or nurse practitioner discussing patient condition. A score of≥7 requires an RRT activation.	Standard care	Not stated (age range 20-85 years)	Not stated	None
Sutherasan (2018) [51]	Thailand	1,145	Hospital (inpatients)	NEWS	On admission, vital signs were used to calculate the NEWS. Patients were classified according to the NEWS at admission as being at low risk (score ≤4), moderate risk (scores 5–6), and high risk (score ≥7). Either essential management or ICU transfer was provided to patients based on the hospital protocol. The protocol was followed by primary care nurses, medical residents, medical fellows, and attending physicians participating in patient care.	Standard care	Adults >18 years who were admitted or transferred to the general medical ward from either the emergency department or ICU.	Patients who were admitted for palliative care only were excluded from this study.	In-hospital mortality ICU admission
Takahashi (2016) [52]	Japan	2,758	Hospital (inpatients on the cardiology ward)	NEWS	NEWS was categorised as a low score (NEWS of 1–4), a medium score (5–6) and a high score (7 or more) and those scores determined the urgency of the clinical response. When scores were more than 5, nurses called cardiologists for the first intervention.	Standard care	Not stated	Not stated	In-hospital mortality Unexpected in-hospital mortality

### Risk of bias

Risk of bias assessments for 37 outcomes in 18 studies are presented in Table 2. Within each study, risk of bias assessments were the same across all outcomes (there was one study [29] for which the domain one classification differed for the two outcomes; however, the overall classification was the same). One study was at low risk of bias, five studies were at moderate risk of bias, and 12 studies were at serious/high risk of bias. The most common reason for bias concerns was not accounting for confounding, particularly in the uncontrolled before-after studies. Many compared different times of year in the two periods (e.g. January–June vs. July–December) leading to a serious risk of bias classification. Issues with missing data and selective reporting (often due to a lack of information) were also common.

**Table 2:**
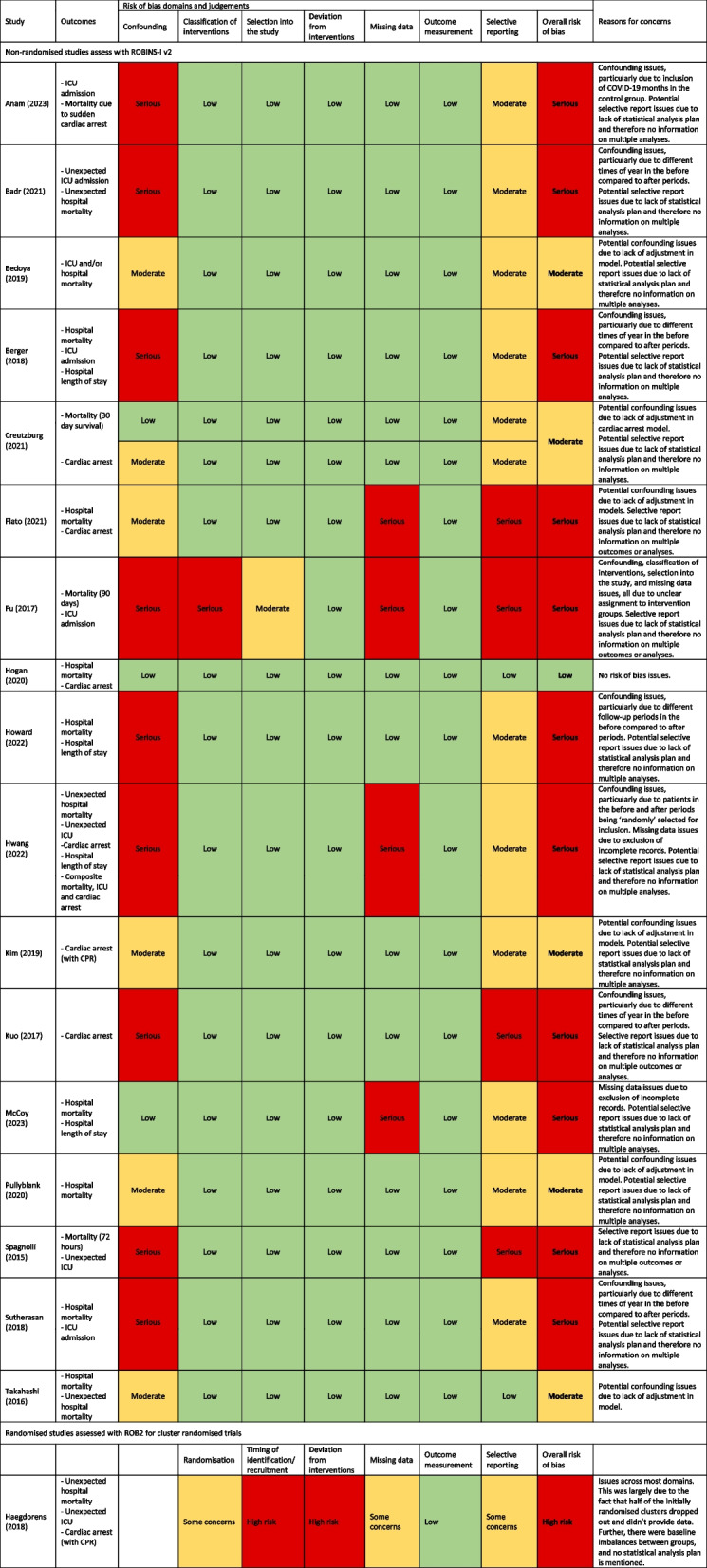
Risk of bias

### Mortality

**Fig. 2:**
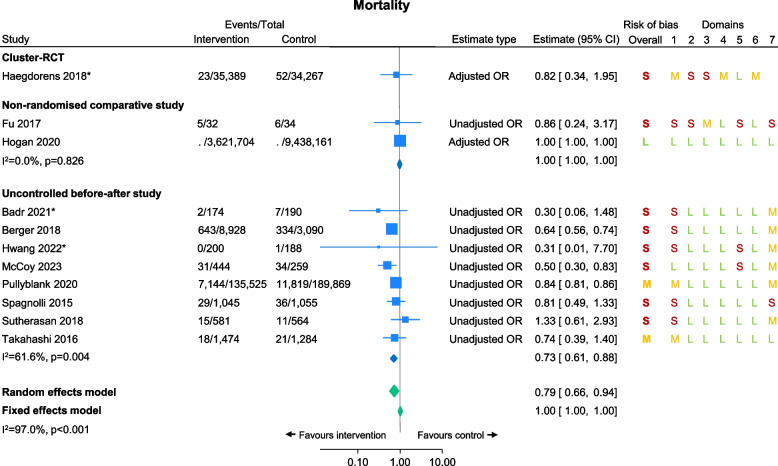
Mortality by study design. *Denotes studies where the outcome was ‘unexpected mortality’ rather than ‘all-cause mortality’. For risk of bias assessments, S=serious risk/high risk, M=moderate risk/some concerns, L=low risk. Note. The Hogan 2020 study presented an adjusted OR of 1.0001 [95% CI 0.9985 to 1.0018] in their paper, which is rounded to 1.00 [95% CI 1.00 to 1.00] in this forest plot. The Takahashi 2016 study also presented unexpected mortality of 5/1474 and 1/1284, unadjusted OR 4.37, 95% CI 0.51 to 37.43; this was excluded from the meta-analysis as it included the same patients as the Takahashi 2016 all-cause mortality outcome. Most studies included patients with a range of conditions admitted to ED, HDU and/or general wards; however, Fu 2017 only includes post-haematopoietic stem cell transplantation (HSCT) patients in a sterile room unit, Pullyblank 2020 only includes patients with suspicion of sepsis codes, and Takahashi 2016 only includes patients on a cardiology ward. Most studies measured ‘in-hospital’ mortality, but Fu 2017 measured 90-day mortality and Spagnolli 2015 measured 72-h mortality

Ten studies reported all-cause mortality as an outcome and four studies reported unexpected mortality; most of these presented in-hospital mortality, but one presented 90-day mortality [31] and one presented 72-h mortality [46]. Eleven studies [25, 28, 31, 34, 39, 43, 45, 46, 51–53] provided enough data to be included in the meta-analysis, including one cluster-RCT, two non-randomised comparative studies, and eight uncontrolled before-after studies (of which one provided an estimate for unexpected mortality as well as all-cause mortality, which is detailed in a footnote; Fig. 2). Low certainty evidence suggested NEWS/NEWS2 reduced mortality (random effect OR 0.79, 95% CI 0.66 to 0.94, *I*^2^=97%; Fig. 2). For the two studies which reported mortality but did not have the data needed for inclusion in the meta-analysis, one was a conference abstract which narratively reported a reduction in in-hospital mortality after implementing the intervention [30] and the other was a full manuscript that presented a *p*-value=0.08 alongside observed/expected means [36].

### Sepsis and suspicion of sepsis

No studies reported sepsis or suspicion of sepsis as an outcome.

### Hospital length of stay

Four studies reported length of stay as an outcome. Berger et al. [28] stated in a conference abstract that it was ‘similar between groups’ and Hwang et al. [39] reported no difference between groups (mean [SD] 5.93 days [5.09] vs. 5.88 days [5.40], *p*=0.937). In contrast, two studies reported a reduction in length of stay in the NEWS/NEWS2 groups: Howard et al. [36] reported a *p*-value=0.004, but did not present means and SDs, and McCoy et al. [43] reported means of 9.17 days in the intervention group vs. 10.54 days in the control group, with a *p*-value=0.027 calculated from an adjusted regression model (effect estimates are not reported).

### ICU admissions

**Fig. 3:**
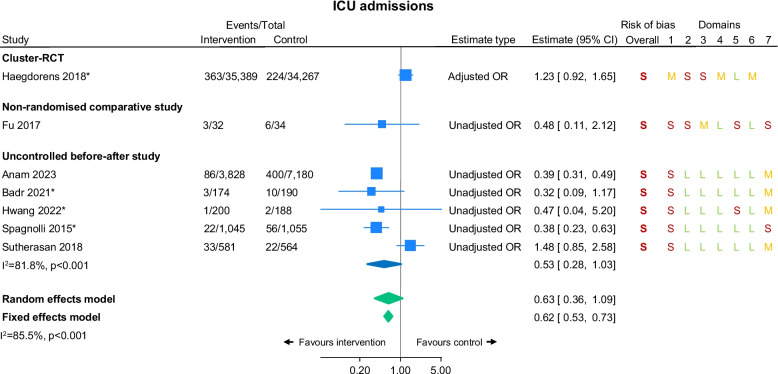
Intensive care unit (ICU) admissions by study design. *Denotes studies where the outcome was ‘unplanned ICU admission’ rather than ‘any ICU admission’. For risk of bias assessments, S=serious risk/high risk, M=moderate risk/some concerns, L=low risk. Note. Most studies included patients with a range of conditions admitted to ED, HDU, and/or general wards; however, Fu 2017 only includes post-haematopoietic stem cell transplantation (HSCT) patients in a sterile room unit

Four studies reported all ICU admissions and a further four studies reported unexpected ICU admissions as outcomes. Seven [23, 25, 31, 39, 46, 51, 53] provided enough data to be included in the meta-analysis, including one cluster-RCT, five uncontrolled before-after studies, and one non-RCT comparative study (Fig. 3). There was low certainty evidence of a potential effect in favour of the NEWS/NEWS2 groups (random effect OR 0.63, 95% CI 0.36 to 1.09, *I*^2^=86%; Fig. 3). The study which was not included in the meta-analysis was a conference abstract stating ICU admissions were ‘similar between groups’ without providing numerical results [28].

### Cardiac arrests

**Fig. 4:**
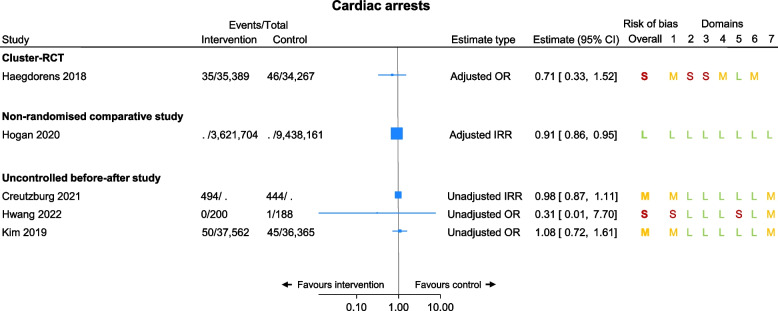
Cardiac arrests by study design. For risk of bias assessments, S=serious concerns, M=moderate concerns, L=low concerns. Note. All studies included patients with a range of conditions admitted to ED, HDU, and/or general wards

This was added as a secondary outcome post hoc as seven of the included studies presented it as an outcome. Of these, five studies are presented in a forest plot (Fig. 4), with four suggesting null results and one large study suggesting a protective effect of using NEWS/NEWS2 (adjusted IRR 0.91, 95% CI 0.86 to 0.95; Fig. 4). These studies have not been meta-analysed due to differing estimate types. Of the two studies excluded from the forest plot, one was a conference abstract [30] which narratively stated a reduction in CA incidence after implementing the intervention, and the other [41] stating ‘the rate of IHCA reduced from 2.53% before intervention, to 2.15% during intervention and to 1.56% after intervention (*p*<0.05)’. In addition, one further study [23] reported in-hospital mortality due to cardiac arrest as an outcome, with results favouring the NEWS2 group (unadjusted OR 0.14, 95% CI 0.08 to 0.24), and a final study [29] reported 30-day mortality following a cardiac arrest with results favouring the NEWS group (adjusted OR 0.61, 95% CI 0.43 to 0.87).

### Composite outcomes

One study [27] reported no difference following implementation of the intervention for a composite outcome of unplanned ICU and/or death, in both their academic facility (adjusted HR 0.94, 95% CI 0.84 to 1.05) and their community hospital (adjusted HR 0.90, 95% CI 0.77 to 1.05). Another study [39] also reported no effect for a composite outcome of hospital mortality, ICU admission and cardiac arrest (1/200 vs. 4/188, *p*=0.203).

### Sub-group analyses

We present subgroup analyses by study design for all meta-analysed outcomes (Figs. 2, 3, and 4). These analyses suggested there was a significant reduction in mortality in the intervention group in the uncontrolled before-after studies but no difference between groups in the cluster-RCT or the non-randomised comparative studies (Fig. 2). For ICU admissions, the uncontrolled before-after studies suggested a potential improvement in the intervention groups, the cluster-RCT suggested a potential improvement in the control group, and the non-randomised comparative study suggested no difference (Fig. 3).

In addition, we present post hoc subgroup analyses by intervention type (Supplemental Figures 1–3). Studies investigating NEWS as part of a package shows a stronger reduction in mortality compared to the control group than studies investigating NEWS or NEWS2 alone (Supplemental Figure 1). For ICU admissions, studies investigating NEWS2 alone show a reduction in ICU admissions (compared to the control group), whereas studies investigating NEWS alone and NEWS as part of a package show no difference between intervention and control groups for ICU admissions (Supplemental Figure 2).

### Sensitivity analyses

There were 15 reported outcomes (7 mortality, 3 ICU admission, 1 cardiac arrest and 2 hospital length of stay) across 8 studies which came from conference abstracts; we explored the impact of removing these from our analyses (Supplemental Figures 4–6). For the mortality meta-analysis, once conference abstracts were removed, there was no improvement due to NEWS/NEWS2, with the random-effects OR dominated by the two large studies (0.91, 95% CI 0.78 to 1.07; Supplemental Figure 4). For ICU admissions, the effect estimate remained similar, but the confidence interval was wider and consistent with both benefit and harm (random effects OR 72, 95% CI 0.36 to 1.45; Supplemental Figure 5). Only one study reporting cardiac arrests was removed so this made little difference to the interpretation of these results (Supplemental Figure 6). Finally, two of the four studies reporting hospital length of stay were removed, one with null findings [28] and one reporting a significant reduction in the NEWS group [43]; again, this made little difference to the conclusions for this outcome.

### Certainty of evidence

Based on GRADE assessments, there is low certainty evidence that NEWS/NEWS2 reduces mortality. There was also a small amount of low certainty evidence that the use of NEWS/NEWS results in a reduction in hospital length of stay and cardiac arrests, and a potential reduction in ICU admissions. All outcomes have been downgraded by two points due to very serious risk of bias concerns (Table 3).

**Table 3:** GRADE assessments

Certainty assessment							№. of patients		Effect		Certainty
№. of studies	Study design	Risk of bias	Inconsistency	Indirectness	Imprecision	Other considerations	Intervention	Control	Relative (95% CI)	Absolute (95% CI)	
**Mortality and unexpected mortality**											
14	Non-randomised studies	Very serious	Not serious	Not serious	Not serious	None	7910/183,492 (4.3%)	12,321/230,800 (5.3%)	**OR 0.79 **(0.66 to 0.94)	11 fewer per 1000 (from 17 fewer to 3 fewer)	⨁⨁◯◯Low
**Hospital length of stay**											
4	Non-randomised studies	Very serious	Not serious	Not serious	Not serious	None	-/9572	-/3537	Not estimable		⨁⨁◯◯Low
**ICU and unexpected ICU admission**											
8	Non-randomised studies	Very serious	Not serious	Not serious	Not serious	None	511/41249 (1.2%)	720/43478 (1.7%)	**OR 0.63 **(0.36 to 1.09)	6 fewer per 1000 (from 11 fewer to 1 more)	⨁⨁◯◯Low
**Cardiac arrest**											
7	Non-randomised studies	Very serious	Not serious	Not serious	Not serious	None	-	-	Not estimable		⨁⨁◯◯Low

## Discussion

### Summary of results

There was low certainty evidence that the use of NEWS/NEWS2 reduced in-hospital mortality, compared with not using NEWS/NEWS2. There was also a small amount of low certainty evidence of a reduction in hospital length of stay and cardiac arrests, and a potential reduction in ICU admissions, when using NEWS/NEWS2. 18/20 studies (90%) investigated NEWS; only 2/20 (10%) investigated NEWS2. All included studies were conducted in hospital settings. Perhaps surprisingly, only two studies (10%) were carried out in the UK. Most of the studies included in the analysis had a serious/high (12/18; 67%) or moderate (5/18; 28%) risk of bias. The sub-group analyses highlighted that the potential evidence for an improvement in mortality and ICU stay were driven by the uncontrolled before-after studies, with no evidence for improvement from the cluster-RCT or non-randomised comparative studies. Similarly, when removing studies only reported in conference abstracts in the sensitivity analyses, potential reductions in mortality and ICU admission from using NEWS/NEWS2 no longer held.

A similar systematic review [55] was published in 2021 exploring the effectiveness of all early warning and rapid response systems in preventing deterioration. Due to their stricter inclusion criteria (which did not include uncontrolled before-after studies), only one of their included studies investigated NEWS; this cluster RCT by Haegdorens et al. [53] is also included in our review. Like our review, all their findings were based on low to very low certainly evidence, and they concluded that given the poor methodological quality of most of the studies, it is not possible to make any recommendations regarding the effectiveness of these early warning systems.

Many studies have explored the predictive accuracy of NEWS/NEWS2 [6–13], and most agree it is a useful tool for detecting patient deterioration. However, several studies have explored the gap between detection of deterioration using early warning scores and subsequent patient management [56–58]. These highlighted that non-adherence to escalation protocols was common [57], and that barriers to appropriate escalation included a lack of standardisation, fear, hierarchical culture, and low staff confidence [56]. Another study found that a facilitation intervention improved escalation as per hospital policy [59]. It is possible then that the reason that the overall body of evidence suggests little improvement in clinical outcomes following the introduction of NEWS/NEWS2 is that the escalation protocols alongside them are not appropriate or are not properly followed. This review further supports this theory, as the subgroup analysis found that studies for which NEWS was introduced as part of a package (which often included standard protocols for rapid response team activation) shows a stronger reduction in mortality (compared to the control group) than studies investigating NEWS or NEWS2 alone.

### Strengths and limitations

To our knowledge, this is the first systematic review looking specifically at the effectiveness of using NEWS/NEWS2 on patient outcomes and provides a comprehensive synthesis of all studies on this topic since the inception of NEWS in 2012. Our inclusion criteria did not impose any limitations by study design or language. We followed Cochrane guidance for conducting systematic reviews, applied GRADE criteria to assess the certainty of the overall body of evidence, and followed PRISMA guidance for reporting. While this review has not identified huge advantages of using NEWS/NEWS2 in terms of clinical outcomes, it has found no evidence at all that the use of NEWS/NEWS2 has a negative impact on clinical outcomes. This is an important finding given their widespread use of NEWS2 across the NHS, particularly in the ambulance service and acute hospitals, and should provide reassurance for its continued use.

There were some limitations in the evidence base. Many of the included studies were conference abstracts, with no associated peer reviewed publication. The data in these studies were limited, and many could not be included in the meta-analyses. Despite multiple attempts to contact many of the authors, only a small number replied and provided data [29, 34, 45]. This resulted in high risk of bias assessments and contributed to the overall low certainty of evidence rating. We explored this limitation in our sensitivity analyses. Most of the included studies evaluated NEWS rather than NEWS2; given this, it was difficult to compare the two (although they are presented separately in subgroup analyses [Supplemental Figures 1–3]). This is unfortunate given that NEWS2 is now widely used rather than the original NEWS. However, several studies have compared NEWS and NEWS2 and found no evidence that NEWS2 is superior [60–62]. We therefore believe these findings may be applicable to both scoring systems.

### Implications for research and/or practice

Based on evidence of its ability to predict clinical outcomes and detect deterioration, NEWS2 has been rolled out across many healthcare settings in England and is mandated by NHS England in acute hospitals and ambulances services [63]. However, this review highlights that there is still further work required to ascertain whether the use of NEWS/NEWS2 leads to an improvement in patient outcomes. In particular, none of the included studies were conducted in the ambulance service, despite their mandated use in this setting, and only one included study was randomised [53]. Because the NEWS/NEWS2 scoring system is now in use in many healthcare settings, particularly hospitals and the ambulance service, there may no longer be the opportunity to conduct randomised controlled trials comparing NEWS2 vs. no NEWS2 in these settings. However, well designed, large-scale non-randomised studies are still possible and should be explored, while being mindful that the way NEWS/NEWS2 is operationalised may differ considerably between hospitals.

### Conclusion

This review highlights a lack of large-scale, high-quality, studies exploring the effectiveness of NEWS/NEWS2. There is some low certainty evidence that the use of NEWS/NEWS2 reduces mortality, but subgroup and sensitivity analyses cast some doubt on this finding. Importantly, no studies reported negative clinical impacts of using NEWS/NEWS2 on any of the outcomes presented in this review, which alongside existing evidence showing the value of NEWS/NEWS2 for detecting patient deterioration, supports its continued use. However, there is still further work required to ascertain whether the NEWS/NEWS2 can improve patient outcomes.

## Supplementary Information

Supplementary Material 1.

Supplementary Material 2.

## Data Availability

Data will be made available on reasonable request. Almost all presented data are publicly available in the original research articles. For data which we requested from authors directly, interested parties would need to contact the authors themselves.

## Abbreviations

CI: Confidence interval
GRADE: Grading of Recommendations Assessment, Development and Evaluation
HR: Hazard ratio
ICU: Intensive care unit
IRR: Incidence rate ratio
MD: Mean difference
NEWS: National Early Warning Score
NEWS2: National Early Warning Score version 2
OR: Odds ratio
RCP: Royal College of Physicians
ROBINS-I: Risk of bias tool for non-randomised studies
ROB2: Risk of bias tool for (cluster) randomised studies
