# Supplementary material for: Does the use of National Early Warning Scores (NEWS or NEWS2) in healthcare settings improve patient outcomes: a systematic review
Source: Syst Rev. 2026 Jan 31;15:73. doi: 10.1186/s13643-026-03088-y (PMC12947470; doi:10.1186/s13643-026-03088-y)
Supplement: Supplementary file 1 — Supplementary Material 1. [file 13643_2026_3088_MOESM1_ESM.docx]

**Search Strategy**

*Search-2 (update 21^st^ October 2024): Summary of numbers:*

- MEDLINE/Embase (29-June-2023 to 21-Oct-2024), n=444
- CINAHL (4-July-2023 to 21-Oct-2024), n=75
- CLib:CENTRAL (4-July-2023 to 21-Oct-2024), n=21
- CLib:CDSR (4-July-2023 to 21-Oct-2024), n=1
- ClinicalTrials.gov (4-July-2023 to 21-Oct-2024), n=11
- WHO-ICTRP (4-July-2023 to 21-Oct-2024), n=3

Total, n= 555

Duplicates removed, n= 52; Records to screen, n=503

*Search-1 (June/July 2023): Summary of numbers:*

- MEDLINE/Embase (Ovid multi-file search) (2012 to 29-June-2023), n=1831
- CINAHL (2012 to 4-July-2023), n=345
- CLib:CENTRAL (2012 to 4-July-2023), n=225 (inc. 141 international trial register records)
- CLib:CDSR (2012 to 4-July-2023), n=6
- ClinicalTrials.gov (all years to 4-July-2023), n=206
- WHO-ICTRP (all years to 4-July-2023), n=30

Total, n=2643

Duplicates removed, n=333; Records to screen, n=2310

*Search strategies:*

Database - Ovid multi-file search

**Embase** <1974 to 30-June-2023; updated 21-October-2024>
**MEDLINE(R) ALL** <1946 to 29-June-2023; updated 21-October-2024>

| 1 | exp NATIONAL EARLY WARNING SCORE/ | 946 |
| --- | --- | --- |
| 2 | (national early warning adj2 (score? or scoring or system?)).mp. | 2488 |
| 3 | (NEWS2 or NEWS-2).tw,kf. | 985 |
| 4 | NEWSS.tw,kf. | 21 |
| 5 | (NEWS* and ((early warning adj2 (score? or scoring or system?)) or EWS)).mp. | 1973 |
| 6 | (NEWS* and (alert* or trigger*) and ((physiological adj2 (measur* or paramet*)) or vital sign? or vitals or (respiration adj2 rat*) or oxygen saturation or (systolic and blood pressure) or pulse rate or consciousness or confusion or temperature or ((critical* or acute*) adj (care or ill* or unwell)) or intensive care or ICU or sepsis)).mp. | 415 |
| 7 | or/1-6 | 3145 |
| 8 | remove duplicates from 7 | 2125 |
| 9 | limit 8 to yr="2012 -Current" | 2052 |
| 10 | *EARLY WARNING SCORE/ | 954 |
| 11 | (((early warning adj2 (score? or scoring or system?)) or EWS) and (emergency or EMS or paramed* or rapid respon* or outpatient? or out-patient? or hospital* or inpatient? or "in-patient?" or EHR or electronic health record?) and ((physiological adj2 (measur* or paramet*)) or vital sign? or vitals or (respiration adj2 rat*) or oxygen saturation or (systolic and blood pressure) or pulse rate or consciousness or confusion or temperature or ((critical* or acute*) adj (care or ill* or unwell)) or intensive care or ICU or sepsis)).mp. | 5216 |
| 12 | (((early warning adj2 (score? or scoring or system?)) or EWS) and (KEWS300* or "KEWS 300*" or "Syncrophi Systems Ltd" or Med eTrax* or MedeTrax* or Patientrack* or Patient track* or SEND or SENDTM or Sensyne Health or CareFlow Vitals* or Care Flow Vitals* or "System C Healthcare" or Vitalpac* or Vital pac* or HL7 or "Health Level 7")).af. | 149 |
| 13 | ((trigger* or alert*) and ((early warning adj2 (score? or scoring or system?)) or EWS)).mp. | 2165 |
| 14 | ("track and trigger" or "track & trigger").mp. | 436 |
| 15 | or/10-14 | 6755 |
| 16 | (systematic review or meta-analysis).ti,pt,hw. | 1215085 |
| 17 | (systematic review? or evidence report* or technology assessment?).jw,jx. | 66729 |
| 18 | (meta-analys* or metaanalys* or meta-synth* or metasynth*).tw,kf. | 731953 |
| 19 | (((systematic* or quantitativ* or methodologic*) adj5 (review* or overview*)) or (systematic* adj3 analys*)).tw,kf. | 922627 |
| 20 | (systematic or structured or evidence or diagnostic or predicti* or trials or studies).ti. and ((review or overview or look or examination or update* or summary).ti. or review.pt.) | 895468 |
| 21 | (quantitativ$ adj5 synthes*).tw,kf. | 14746 |
| 22 | ((research adj3 (integrati* or overview*)) or (integrative adj2 review*) or research integration).tw,kf. | 32182 |
| 23 | scoping review?.ti,kf. or (review.ti,kf,pt. and (trials as topic or studies as topic).hw.) | 224264 |
| 24 | ((diagnostic or evidence) adj3 review*).tw,kf. | 174209 |
| 25 | review.pt. and (medline or medlars or embase or pubmed or scisearch or psychinfo or psycinfo or psychlit or psyclit or cinahl or electronic database* or bibliographic database* or computeri#ed database* or online database* or pooling or pooled or mantel haenszel or peto or dersimonian or der simonian or fixed effect or ((hand adj2 search*) or (manual* adj2 search*))).tw,kf,hw. | 522955 |
| 26 | (technology assessment* or HTA or HTAs or technology overview* or technology appraisal*).tw,kf. | 32983 |
| 27 | (0266-4623 or 1469-493X or 1366-5278 or 1530-440X or 2046-4053).is. | 47645 |
| 28 | (literature review or mixed method? or rapid review or "review of reviews" or scoping review or umbrella review).tw,kf. | 518813 |
| 29 | or/16-28 | 2447915 |
| 30 | 15 and 29 | 484 |
| 31 | remove duplicates from 30 | 309 |
| 32 | (advisory committee or advisory group or guideline? or consensus statement or international consensus).ti,kf. | 297393 |
| 33 | 15 and 32 | 50 |
| 34 | remove duplicates from 33 | 36 |
| 35 | 31 or 34 | 336 |
| 36 | limit 35 to yr="2012 -Current" | 312 |
| 37 | 9 or 36 | 2292 |
|  | Remaining records: S1 = 1831; S2 = 444  (after additional duplicates removed from this multi-file search) |  |

**Database – CINAHL** (EBSCOhost) S1 04/07/2023; S2 update 21/10/2024

Advanced Search

| \| **#** \| **Query** \| **Results** \| \| --- \| --- \| --- \| \| S32 \| S6 OR S29 Exclude MEDLINE  (75 new (Oct 2024)) \| 345  [381] \| \| S31 \| S6 OR S29 Published Date: 20120101-20231231; -20241231 \| 565 [613] \| \| S30 \| S6 OR S29 \| 585 [634] \| \| S29 \| S12 AND S28 \| 157 [185] \| \| S28 \| S13 OR S14 OR S15 OR S16 OR S17 OR S18 OR S19 OR S20 OR S21 OR S22 OR S23 OR S24 OR S25 OR S26 OR S27 \| 578,815 [615,093] \| \| S27 \| SU ( (“advisory committee” or “advisory group” or “advisory groups” or guideline* or “consensus statement” or “international consensus”) ) OR AB ( (“advisory committee” or “advisory group” or “advisory groups” or guideline* or “consensus statement” or “international consensus”) ) \| 225,478 [229,422] \| \| S26 \| SU guideline* \| 96,728 [100,742] \| \| S25 \| TI(Review) and AB(review*) \| 143,178 [151,510] \| \| S24 \| TI( “literature review” or “mixed method” or “mixed methods” or “rapid review” or "review of reviews" or “scoping review” or “umbrella review” ) OR AB ( “literature review” or “mixed method” or “mixed methods” or “rapid review” or "review of reviews" or “scoping review” or “umbrella review” ) \| 93,902 [98,684] \| \| S23 \| MH(literature review) \| 8,978 [9,439] \| \| S22 \| IS "0266-4623" or "1469-493X" or "1366-5278" or "1530-440X" or "2046-4053" \| 9,683 [10,035] \| \| S21 \| (TI(review) or SH(review)) and AB(medline or medlars or embase or pubmed or scisearch or psychinfo or psycinfo or psychlit or psyclit or cinahl or “electronic database” or “electronic databases” or “bibliographic database” or “bibliographic databases” or “computerized database” or “computerized databases” or “computerised database” or “computerised databases” or “online database” or “online databases” or pooling or pooled or “mantel Haenszel” or peto or dersimonian or “der simonian” or “fixed effect” or (hand N2 search*) or (manual* N2 search*)) \| 82,019 [90,945] \| \| S20 \| TI ( ((diagnostic or evidence) N3 review*) ) OR AB ( ((diagnostic or evidence) N3 review*) ) \| 34,952 [36,434] \| \| S19 \| TI ( ((research N3 (integrati* or overview*)) or (integrative N2 review*) or “research integration”) ) OR AB ( ((research N3 (integrati* or overview*)) or (integrative N2 review*) or “research integration”) ) \| 11,540 [12,561] \| \| S18 \| TI (quantitative* N5 synthes*) OR AB (quantitative* N5 synthes*) \| 1,797 [1,964] \| \| S17 \| TI ( “technology assessment” or “technology assessments” or HTA or HTAs or “technology overview” or “technology overviews” or “technology appraisal” or “technology appraisals” ) OR AB ( “technology assessment” or “technology assessments” or HTA or HTAs or “technology overview” or “technology overviews” or “technology appraisal” or “technology appraisals” ) \| 4,657 [4,823] \| \| S16 \| TI(systematic or structured or evidence or diagnostic or predicti* or trials or studies) and (TI (review or overview or look or examination or update* or summary) or sh(review)) \| 137,323 [158,096] \| \| S15 \| TI ( (((systematic* or quantitativ* or methodologic*) N5 (review* or overview*)) or (systematic* N3 analys*)) ) OR AB ( (((systematic* or quantitativ* or methodologic*) N5 (review* or overview*)) or (systematic* N3 analys*)) ) \| 161,127 [181,577] \| \| S14 \| TI ( meta-analys* or metaanalys* or meta-synth* or metasynth or “evidence report” or “evidence reports” ) OR AB ( meta-analys* or metaanalys* or meta-synth* or metasynth or “evidence report” or “evidence reports") \| 108,745 [122,914] \| \| S13 \| (MH "Systematic Review") OR (MH "Meta Analysis") OR (MH "Meta Synthesis") \| 154,031 [169,973] \| \| S12 \| S7 OR S8 OR S9 OR S10 OR S11 \| 1,074 [1,212] \| \| S11 \| TI ( ("track and trigger" or "track & trigger") ) OR AB ( ("track and trigger" or "track & trigger") ) \| 96 [94] \| \| S10 \| TI ( ((trigger* or alert*) and ("early warning" or EWS)) ) OR AB ( ((trigger* or alert*) and ("early warning" or EWS)) ) \| 373 [376] \| \| S9 \| (MH "Early Warning Score") \| 181 [323] \| \| S8 \| TI ( (((“early warning” or EWS) and (KEWS300 or KEWS300TM OR KEWS300R or “KEWS 300” or “KEWS 300TM” or “KEWS 300R” or "Syncrophi Systems Ltd" or MedeTrax or MedeTraxTM or MedeTraxR or “Med eTrax” or “Med eTraxTM” or “Med eTraxR” or Patientrack or PatientrackTM or PatientrackR or “Patient track” or “Patient trackTM” or “Patient trackR” or SEND or SENDTM or SENDR or “Sensyne Health” or CareFlow or “Care Flow” or "System C Healthcare" or Vitalpac or VitalpacTM or VitalpacR or “Vital pac” or “vital pacTM” or “vital pacR” or HL7 or "Health Level 7")) ) OR AB ( (((“early warning” or EWS) and (KEWS300 or KEWS300TM OR KEWS300R or “KEWS 300” or “KEWS 300TM” or “KEWS 300R” or "Syncrophi Systems Ltd" or MedeTrax or MedeTraxTM or MedeTraxR or “Med eTrax” or “Med eTraxTM” or “Med eTraxR” or Patientrack or PatientrackTM or PatientrackR or “Patient track” or “Patient trackTM” or “Patient trackR” or SEND or SENDTM or SENDR or “Sensyne Health” or CareFlow or “Care Flow” or "System C Healthcare" or Vitalpac or VitalpacTM or VitalpacR or “Vital pac” or “vital pacTM” or “vital pacR” or HL7 or "Health Level 7")) ) \| 24 [22] \| \| S7 \| TI ( (((“early warning” N2 (score* or scoring or system*)) or EWS) AND (emergency or EMS or paramed* or “rapid response” or “rapid responder” or “rapid responders” or outpatient* or out-patient* or hospital* or inpatient* or "in-patient" or "in-patients" or EHR or “electronic health record” or “electronic health records”) AND (physiological or vital or vitals or respiration or “oxygen saturation” or “blood pressure” or “pulse rate” or consciousness or confusion or temperature or critical* or “intensive care” or ICU or sepsis)) ) OR AB ( (((“early warning” N2 (score* or scoring or system*)) or EWS) AND (emergency or EMS or paramed* or “rapid response” or “rapid responder” or “rapid responders” or outpatient* or out-patient* or hospital* or inpatient* or "in-patient" or "in-patients" or EHR or “electronic health record” or “electronic health records”) AND (physiological or vital or vitals or respiration or “oxygen saturation” or “blood pressure” or “pulse rate” or consciousness or confusion or temperature or critical* or “intensive care” or ICU or sepsis)) ) \| 681 [703] \| \| S6 \| S1 OR S2 OR S3 OR S4 OR S5 \| 488 [493] \| \| S5 \| TI ( (NEWS* and (alert* or trigger*) and ((physiological N2 (measur* or paramet*)) or “vital sign” or “vital signs” or vitals or (respiration N2 rat*) or “oxygen saturation” or (systolic and “blood pressure”) or “pulse rate” or consciousness or confusion or temperature or ((critical* or acute*) N1 (care or ill* or unwell)) or “intensive care” or ICU or sepsis)) ) OR AB ( (NEWS* and (alert* or trigger*) and ((physiological N2 (measur* or paramet*)) or “vital sign” or “vital signs” or vitals or (respiration N2 rat*) or “oxygen saturation” or (systolic and blood pressure) or “pulse rate” or consciousness or confusion or temperature or ((critical* or acute*) N1 (care or ill* or unwell)) or “intensive care” or ICU or sepsis)) ) \| 51 [46] \| \| S4 \| TI ( (NEWS* and (("early warning" N2 (score* or scoring or system*)) or EWS)) ) OR AB ( (NEWS* and (("early warning" N2 (score* or scoring or system*)) or EWS)) ) \| 304 [310] \| \| S3 \| TI NEWSS OR AB NEWSS \| 3 [3] \| \| S2 \| TI ( NEWS2 or "NEWS-2" ) OR AB ( NEWS2 or "NEWS-2" ) \| 94 [111] \| \| S1 \| TI ( ("national early warning" N2 (score* or scoring or system*)) ) OR AB ( ("national early warning" N2 (score* or scoring or system*)) ) \| 404 [423] \| |
| --- | --- | --- | --- | --- | --- | --- | --- | --- | --- | --- | --- | --- | --- | --- | --- | --- | --- | --- | --- | --- | --- | --- | --- | --- | --- | --- | --- | --- | --- | --- | --- | --- | --- | --- | --- | --- | --- | --- | --- | --- | --- | --- | --- | --- | --- | --- | --- | --- | --- | --- | --- | --- | --- | --- | --- | --- | --- | --- | --- | --- | --- | --- | --- | --- | --- | --- | --- | --- | --- | --- | --- | --- | --- | --- | --- | --- | --- | --- | --- | --- | --- | --- | --- | --- | --- | --- | --- | --- | --- | --- | --- | --- | --- | --- | --- | --- | --- | --- | --- |

**Cochrane Library**, issue 7 of 12, 2023

Date Run: S1 04/07/2023; S2 update 21/10/2024

| ID | Search | Hits |
| --- | --- | --- |
| #1 | ("national early warning" NEAR/2 (score* or scoring or system*)):ti,ab,kw | 152 [167] |
| #2 | ("NEWS2" or "NEWS-2"):ti,ab,kw | 93 [104] |
| #3 | (NEWSS):ti,ab,kw | 0 |
| #4 | (NEWS* and (("early warning" NEAR/2 (score* or scoring or system*)) or EWS)):ti,ab,kw | 128 [144] |
| #5 | (NEWS* and (alert* or trigger*) and ((physiological NEAR/2 (measur* or paramet*)) or vital or vitals or respiration or "oxygen saturation" or "blood pressure" or "pulse rate" or consciousness or confusion or temperature or critical* or "intensive care" or ICU or sepsis)):TI,AB,KW | 44 [45] |
| #6 | ((“early warning” or EWS) and (KEWS300 or KEWS300TM OR KEWS300R or “KEWS 300” or “KEWS 300TM” or “KEWS 300R” or "Syncrophi Systems Ltd" or MedeTrax or MedeTraxTM or MedeTraxR or “Med eTrax” or “Med eTraxTM” or “Med eTraxR” or Patientrack or PatientrackTM or PatientrackR or “Patient track” or “Patient trackTM” or “Patient trackR” or SEND or SENDTM or "SENDR" or “Sensyne Health” or CareFlow or “Care Flow” or "System C Healthcare" or Vitalpac or VitalpacTM or VitalpacR or “Vital pac” or “vital pacTM” or “vital pacR” or HL7 or "Health Level 7")):ti,ab,kw | 5 |
| #7 | ("track and trigger" or "track & trigger"):ti,ab,kw | 15 [16] |
| #8 | (#1 OR #2 OR #3 OR #4 OR #5 OR #6 OR #7) | 238 [256] |
| Limit to CENTRAL (Trials), 01/01/2012 to 05/07/2023; 21/10/2024 (21 new) | | 225 [244 >2012] |
| #9 | (((“early warning” NEAR/2 (score* or scoring or system*)) or EWS) AND (emergency or EMS or paramed* or “rapid response” or “rapid responder” or “rapid responders” or outpatient* or out-patient* or hospital* or inpatient* or "in-patient" or "in-patients" or EHR or “electronic health record” or “electronic health records”) AND (physiological or vital or vitals or respiration or “oxygen saturation” or “blood pressure” or “pulse rate” or consciousness or confusion or temperature or critical* or “intensive care” or ICU or sepsis)):ti,ab,kw | 200 [220] |
| #10 | "Early Warning Score":kw | 57 [69] |
| #11 | ((trigger* or alert*) and ("early warning" or EWS)):ti,ab,kw | 92 [98] |
| #12 | #9 OR #10 OR #11 | 266 [294] |
| Limit to CDSR, 01/01/2012 to 05/07/2023; 21/10/2024 (1 new) | | 6 [3] |

**International Trial Registers**

| **ClinicalTrials.gov**  <https://clinicaltrials.gov/> | **WHO-ICTRP**  <https://trialsearch.who.int/> |
| --- | --- |
| Search-1 (04/07/2023) | |
| #1 “national early warning” (172)  #2 NEWS2 OR "NEWS-2" OR NEWSS (112)  #3 (#1 OR #2) (206) | #1 national early warning (23)  #2 NEWS2 OR "NEWS-2" OR NEWSS (7)  #3 (#1 OR #2) (30) |
| Search-2 (update, 21/10/2024) | |
| #1 “national early warning” (180)  #2 NEWS2 OR "NEWS-2" OR NEWSS (133)  #3 (#1 OR #2) (215) [11 new] | #1 national early warning (26)  #2 NEWS2 OR "NEWS-2" OR NEWSS (6)  #3 (#1 OR #2) (31) [3 New] |

**********************************************************************************

**Supplemental Figure 1: Mortality by intervention type**

**
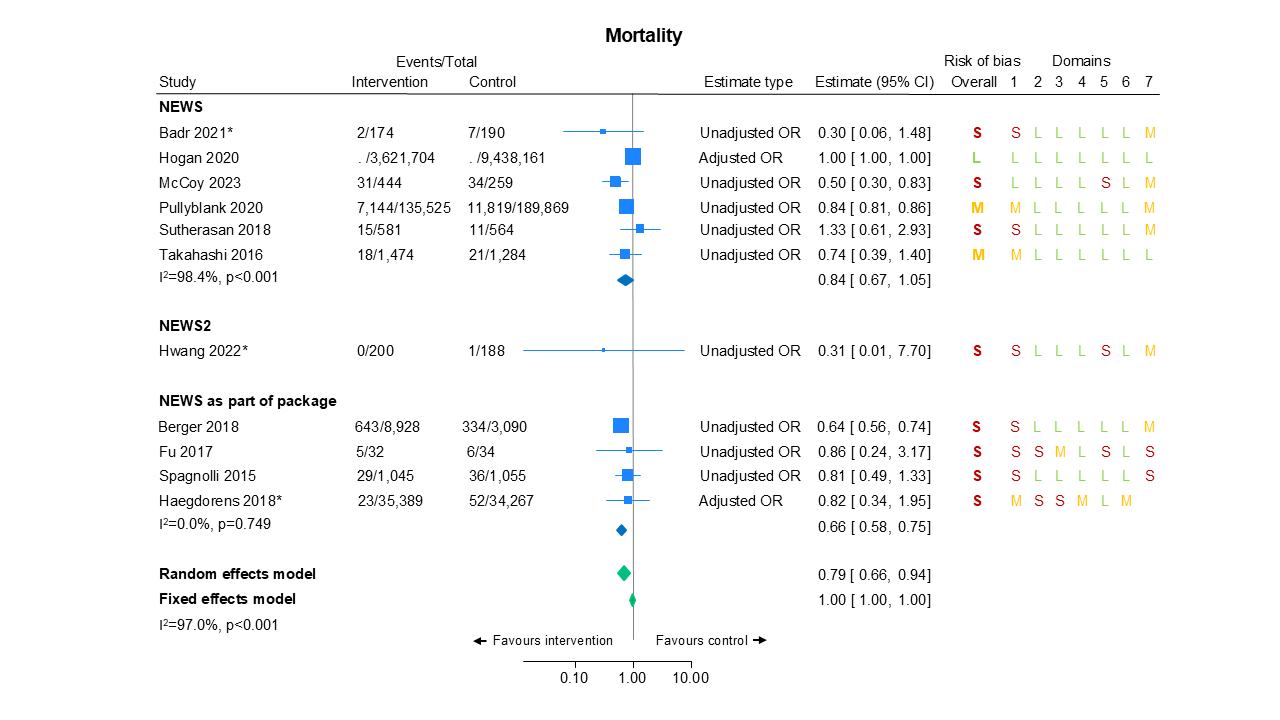
**

* Denotes studies where the outcome was ‘unexpected mortality’ rather than ‘all-cause mortality’.

For risk of bias assessments, S=Serious risk/ High risk, M=Moderate risk/ Some concerns, L=Low risk.

Notes. The Hogan 2020 study presented an adjusted OR of 1.0001 [95% CI 0.9985 to 1.0018] in their paper, which is rounded to 1.00 [95% CI 1.00 to 1.00] in this forest plot. The Takahashi 2016 study also presented unexpected mortality of 5/1474 and 1/1284, unadjusted OR 4.37, 95% CI 0.51 to 37.43; this was excluded from the meta-analysis as it included the same patients as the Takahashi 2016 all-cause mortality outcome. Most studies included patients with a range of conditions admitted to ED, HDU and/or general wards; however, Fu 2017 only includes post hematopoietic stem cell transplantation (HSCT) patients in a sterile room unit, and Takahashi 2016 only includes patients on a cardiology ward.

**Supplemental Figure 2: ICU admissions by intervention type**


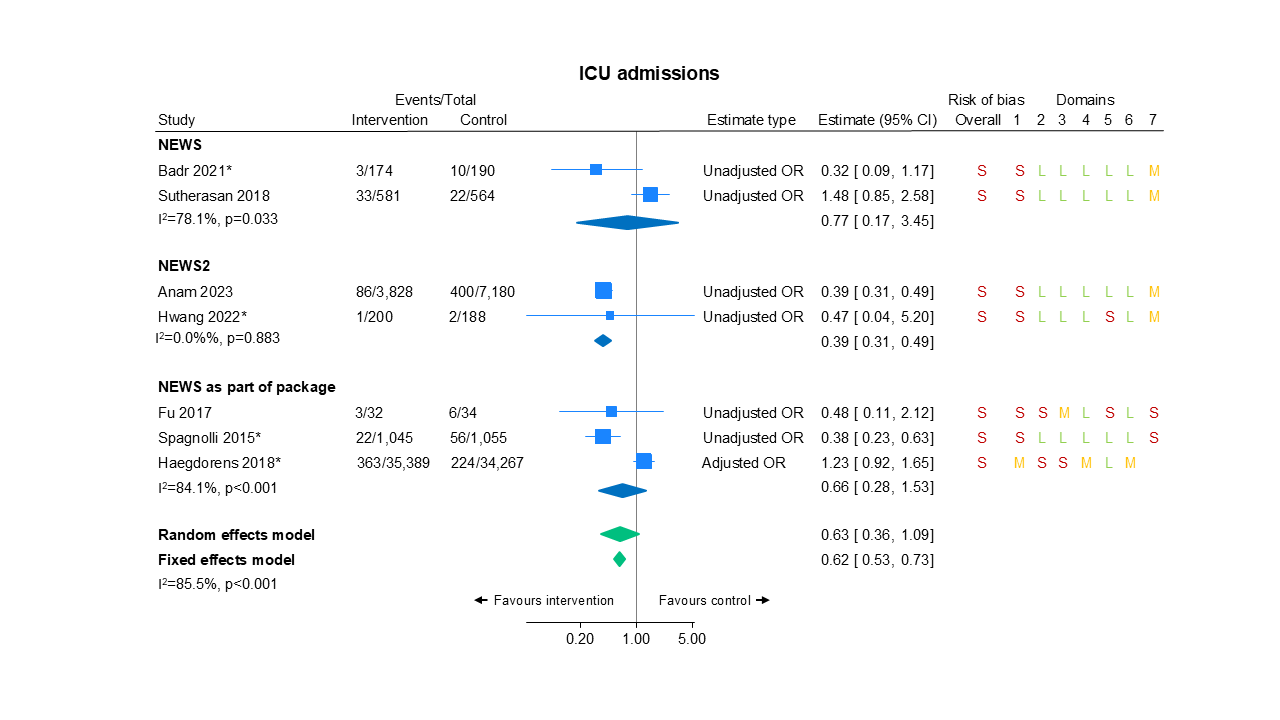


* Denotes studies where the outcome was ‘unplanned ICU admission’ rather than ‘any ICU admission’.

For risk of bias assessments, S=Serious risk/ High risk, M=Moderate risk/ Some concerns, L=Low risk.

Notes. Most studies included patients with a range of conditions admitted to ED, HDU and/or general wards; however, Fu 2017 only includes post hematopoietic stem cell transplantation (HSCT) patients in a sterile room unit.

**Supplemental Figure 3: Cardiac arrests by intervention type**

**
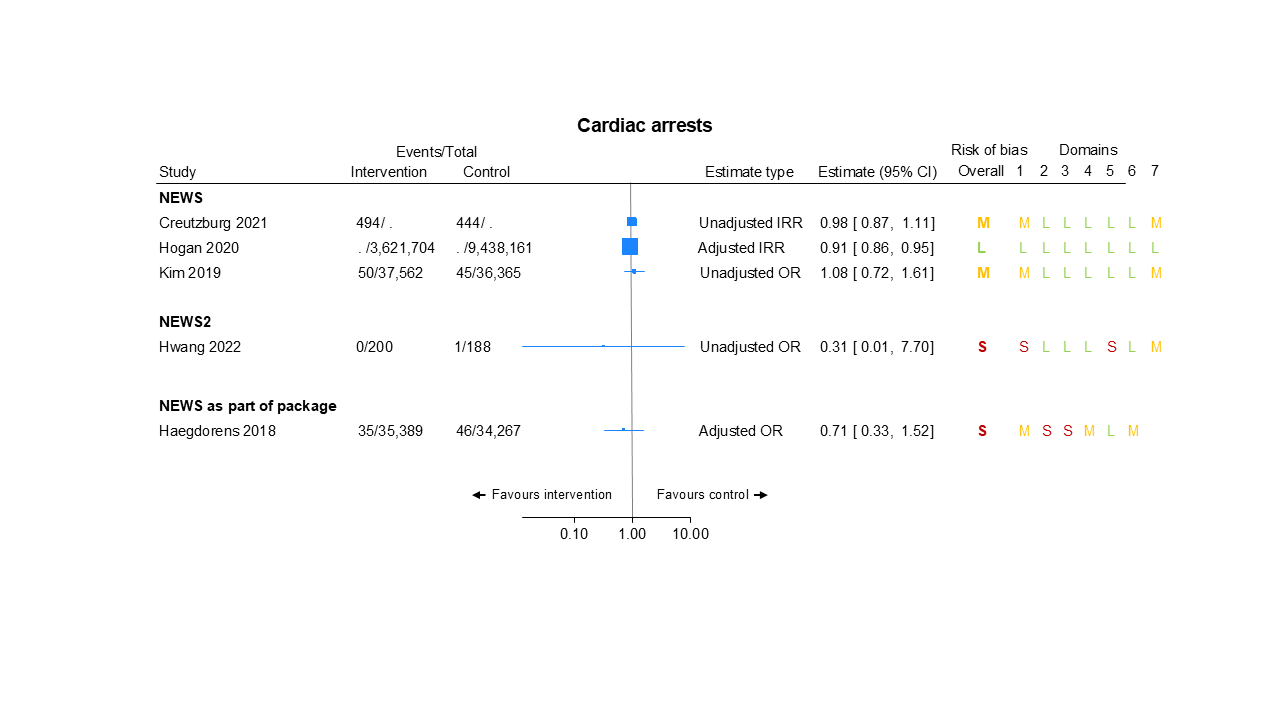
**

For risk of bias assessments, S=Serious risk/ High risk, M=Moderate risk/ Some concerns, L=Low risk.

Notes. All studies included patients with a range of conditions admitted to ED, HDU and/or general wards.

**Supplemental Figure 4: Mortality by study design – excluding conference abstracts**

**
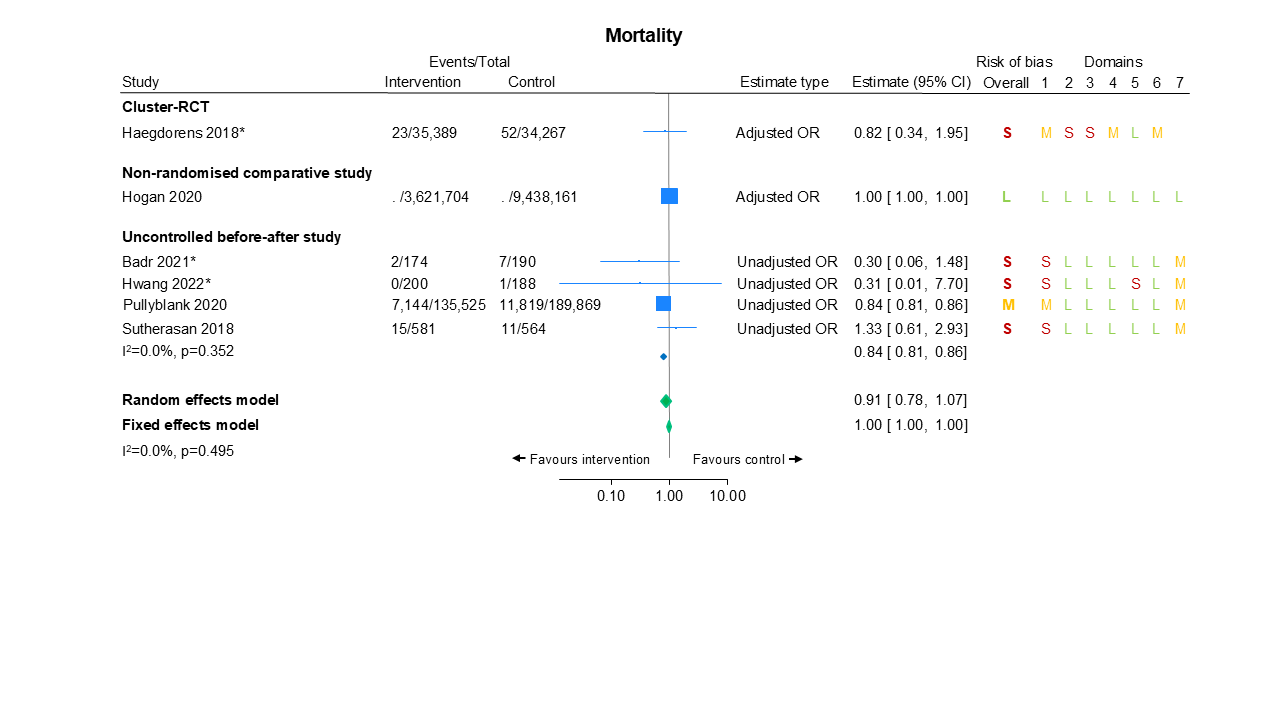
**

* Denotes studies where the outcome was ‘unexpected mortality’ rather than ‘all-cause mortality’.

For risk of bias assessments, S=Serious risk/ High risk, M=Moderate risk/ Some concerns, L=Low risk.

Notes. The Hogan 2020 study presented an adjusted OR of 1.0001 [95% CI 0.9985 to 1.0018] in their paper, which is rounded to 1.00 [95% CI 1.00 to 1.00] in this forest plot. All studies included patients with a range of conditions admitted to ED, HDU and/or general wards.

**Supplemental Figure 5: ICU admissions by study design – excluding conference abstracts**


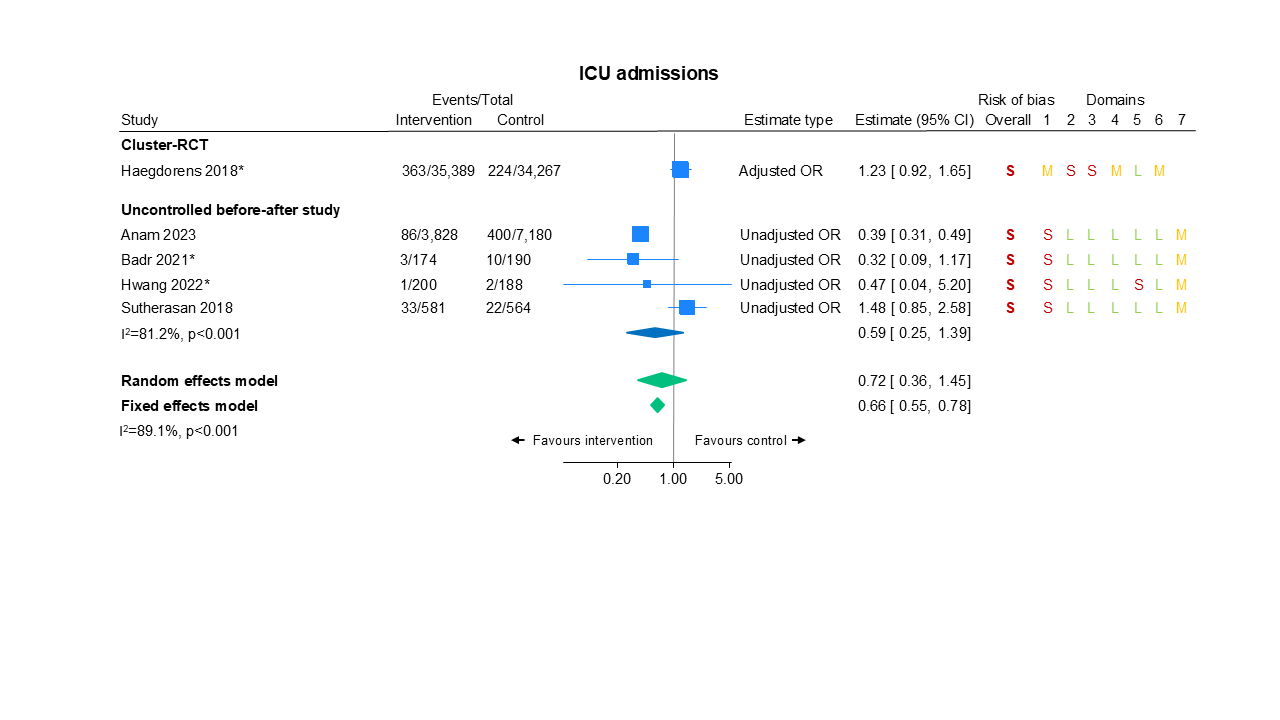
* Denotes studies where the outcome was ‘unplanned ICU admission’ rather than ‘any ICU admission’.

For risk of bias assessments, S=Serious risk/ High risk, M=Moderate risk/ Some concerns, L=Low risk.

Notes. All studies included patients with a range of conditions admitted to ED, HDU and/or general wards.

**Supplemental Figure 6: Cardiac arrests by study design – excluding conference abstracts**


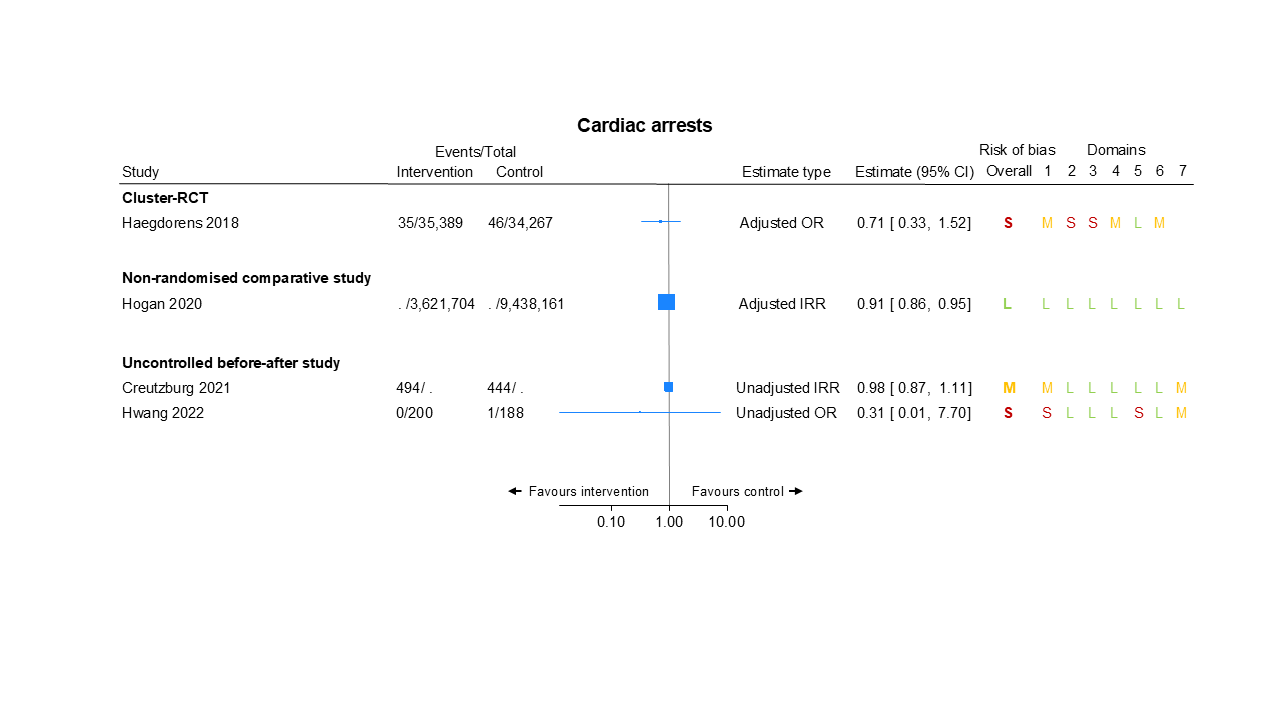


For risk of bias assessments, S=Serious risk/ High risk, M=Moderate risk/ Some concerns, L=Low risk.

Notes. All studies included patients with a range of conditions admitted to ED, HDU and/or general wards.
